# Colour-crafted phosphor-free white light emitters via *in-situ* nanostructure engineering

**DOI:** 10.1038/srep44148

**Published:** 2017-03-08

**Authors:** Daehong Min, Donghwy Park, Kyuseung Lee, Okhyun Nam

**Affiliations:** 1 Convergence Center for Advanced Nano Semiconductors (CANS), Department of Nano-Optical Engineering, Korea Polytechnic University, 237, Sangidaehak-ro, Siheung-si, Gyeonggi-do 429-793, South Korea

## Abstract

Colour-temperature (T_c_) is a crucial specification of white light-emitting diodes (WLEDs) used in a variety of smart-lighting applications. Commonly, T_c_ is controlled by distributing various phosphors on top of the blue or ultra violet LED chip in conventional phosphor-conversion WLEDs (PC-WLEDs). Unfortunately, the high cost of phosphors, additional packaging processes required, and phosphor degradation by internal thermal damage must be resolved to obtain higher-quality PC-WLEDs. Here, we suggest a practical *in-situ* nanostructure engineering strategy for fabricating T_c_-controlled phosphor-free white light-emitting diodes (PF-WLEDs) using metal-organic chemical vapour deposition. The dimension controls of *in-situ* nanofacets on gallium nitride nanostructures, and the growth temperature of quantum wells on these materials, were key factors for T_c_ control. Warm, true, and cold white emissions were successfully demonstrated in this study without any external processing.

In the early days of light-emitting diode (LED) technology, increasing the optical power and reducing production costs were the most crucial issues for conventional phosphor-conversion white LEDs (PC-WLEDs). Today however, the colour temperature (Tc) is also important specifications of LEDs for use in advanced lighting applications such as emotional lighting and smart lighting systems^1^. In general T_c_ is controlled by using various types of phosphor materials, which are distributed on the blue or ultra violet (UV) LED chip during the LED packaging process^2^,^3^,^4^. However, these materials are quite expensive and additional phosphor distribution is needed in PC-WLED manufacturing when they are used. Moreover, these phosphor materials gradually deteriorate due to the accumulated heat in the PC-WLED^5^ during operation. As a result, T_c_ is unintentionally changed by decreasing the light conversion efficiency of the phosphors, resulting in blue-shifted white emissions. Many research groups have conducted work on phosphor-free white light-emitting diodes (PF-WLEDs), as these LEDs feature minimal T_c_ variation from phosphor degradation as well as lower manufacturing costs. There are many current technical approaches to fabricate PF-WLEDs, such as indium gallium nitride (InGaN) based layer-by-layer growth (utilizing a different indium composition in each active layer^6^,^7^,^8^), or InGaN quantum dots (QDs) embedded within InGaN layer structures^9^,^10^. Unfortunately, these methods have not yet been commercialized, due to the low quality and insufficient optical power of the In-rich InGaN layers. To address this issue, some groups have suggested using a three-dimensional (3D) GaN structure method. In this method, InGaN active layers are grown as a 3D GaN structure, with the indium incorporation and thickness of the InGaN layers changing along the GaN multifacets. Broad light emission was demonstrated as a result of this method^11^,^12^,^13^,^14^. However, an additional photolithography process is necessary to form an external dielectric mask such as silicon dioxide (SiO_2_) in selective area growth (SAG). Moreover, a variety of pattern masks are also required to control the T_c_ of PF-WLEDs, as the emission wavelength is controlled by the shapes of the multfacets^15^,^16^. This is a critical obstacle for device development, due to requiring multi-step processing and increased fabrication costs cancelling out the technical advantages compared to conventional phosphor methods. To overcome this issue, we previously reported an *in-situ* silicon nitride (SiN_x_) nanomask technique to form high-density nano-sized 3D GaN multifacets with defect blocking in an epi-structured PF-WLED; as a result, a broad white emission was successfully demonstrated using electroluminescence (EL) measurements^17^. Thereafter, we attempted colour control of the PF-WLED by *in-situ* processing. In this study, we suggest colour-crafted PF-WLEDs for T_c_ control using *in-situ* nanostructure engineering for the first time. InGaN/GaN multiple quantum wells (MQWs) were grown on these nanostructures as active layers. In our study the SiN_x_ nanomask density and MQWs growth temperature were the main factors for altering T_c_ of the PF-WLEDs. As a result, warm/true/cold white PF-WLEDs were successfully demonstrated.

## Results and Discussion

### *In-situ* SiN_x_ nanomask layer deposition

**Figure 1:**
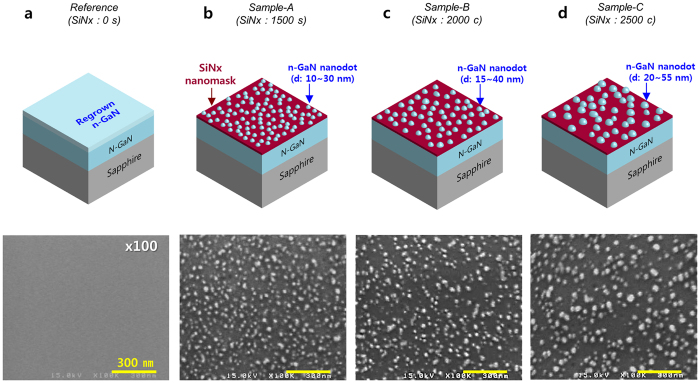
Schematics and SEM surface images of four regrown n-GaN templates. (**a**) Reference (without SiN_x_ nanomask). (**b**) Sample-A (SiN_x_ deposition time: 1500 s). (**c**) Sample-B (SiN_x_ deposition time: 2000 s). (**d**) Sample-C (SiN_x_ deposition time: 2500 s). The regrown n-GaN density on sample-A, sample-B, and sample-C is 4.7 × 10^10^/cm^2^, 3.7 × 10^10^/cm^2^, and 2.9 × 10^10^/cm^2^, respectively.

To investigate the effects of the *in-situ* SiN_x_ nanomask deposition, we grew n-type doped GaN (n-GaN) templates on a c-plane sapphire substrate using metal-organic chemical vapour deposition (MOCVD). The *in-situ* SiN_x_ nanomask layers were deposited on the n-GaN templates for variable time lengths. These experimental conditions are further detailed in the “Methods” sections. Finally, the n-GaN layer was regrown at 850 °С for 3 s without coalescence of the n-GaN nanostructure with each other. As shown in the scanning electron microscopy (SEM) images of Fig. 1, the four templates show different surface morphologies depending on the SiN_x_ nanomask deposition time. These templates were labelled based on deposition time as reference (0 s), sample-A (1500 s), sample-B (2000 s), and sample-C (2500 s). As shown in Fig. 1, while the reference (no SiN_x_ nanomask) features a flat surface, samples-A, B, and C feature high-density n-GaN nanodots (G.D.). These results confirm that the regrown n-GaN layer was selectively grown on the open sites of the *in-situ* SiN_x_ nanomask. Moreover, samples-A, B, and C have different n-GaN nanodot densities of 4.7 × 10^10^/cm^2^, 3.7 × 10^10^/cm^2^, and 2.9 × 10^10^/cm^2^, respectively. These results indicate that the density of openings on the SiNx nanomask changes based on deposition time. The size of the n-GaN nanodots on each sample is inversely proportional to the opening density of the corresponding nanomask. Sample-A features nanodots approximately 10–30 nm in diameter, with the narrowest nanodot spacing among the three samples. Sample-B and sample-C show nanodot diameters of approximately 15–40 nm and 20–55 nm, respectively. Sample-C shows that the average size of n-GaN nanodots is the largest among these samples, indicating that more Ga adatoms were incorporated into the open areas of the SiN_x_ nanomask layer than in sample-A and sample-B. As a result, we could control the size and the distribution density of the n-GaN nanodots by changing the deposition time of the SiN_x_ nanomask.

### 3D n-type GaN nanostructure growth

**Figure 2:**
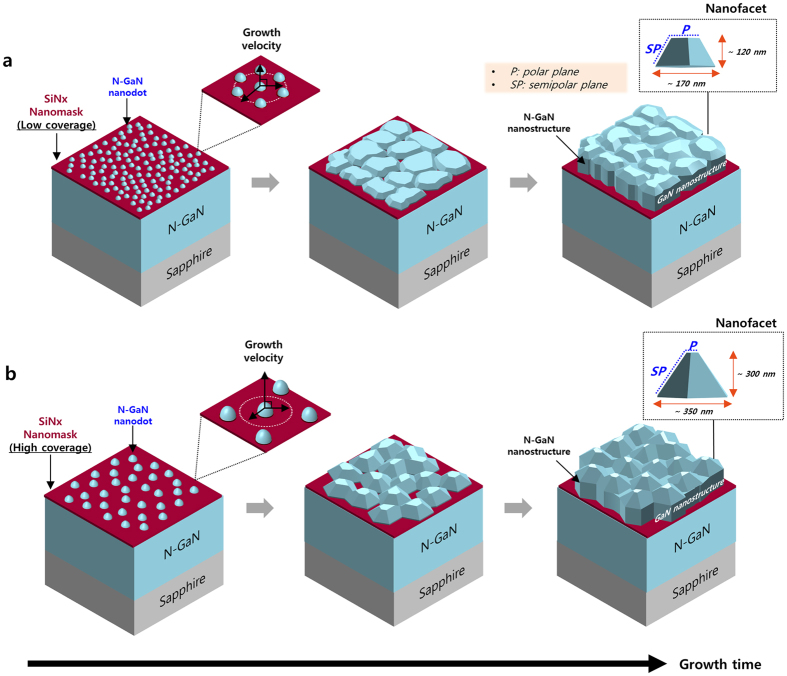
Schematics of 3D n-GaN nanostructure growth with SiN_x_ nanomask coverage. (**a**) Regrowth process of n-GaN nanostructures with low-coverage SiN_x_ nanomask template (sample-A). (**b**) Regrowth process of n-GaN nanostructures with high-coverage SiN_x_ nanomask template (sample-C). The grey-spot-line squares show the shape differences of the n-GaN nanostructures, depending on SiN_x_ nanomask coverage. P and SP represents the polar plane of GaN (such as (0001)) and semipolar planes of GaN (such as {11–22} or {10–11}), respectively.

To fabricate the n-GaN nanostructures, we re-grew the 3D n-GaN structure on *in-situ* SiN_x_ nanomask-embedded templates for 600 s at 850 °С. Figure 2 shows a schematic of the growth process for the 3D n-GaN nanostructures. As shown in Fig. 2, the shape of the n-GaN nanostructure surface depends strongly on the coverage of the SiNx nanomask. For SiN_x_ nanomasks with low coverage (sample-A), the vertical growth rate is relatively slower than that of high coverage nanomasks (sample-C) because many of the neighbouring n-GaN nanodots are laterally merged with each other. This accelerates the formation of the low trapezoidal shape of the n-GaN nanostructure with flat top-plane [P: polar plane (0002) on GaN] and inclined-plane [SP: semipolar planes {11–22} or {10–11} of GaN] surfaces. On the other hand, the shape of the n-GaN nanostructure grown on high coverage SiN_x_ nanomask (sample-C) template features a narrower top-plane than that of the low-coverage SiN_x_ template, due to the fast growth rate of n-GaN due to the low density of openings on this SiN_x_ nanomask (Fig. 2(b)). To confirm these changes of 3D nanostructures, we conducted additional experiments of 3D n-GaN nanostructures grown at higher growth temperatures and shorter growth times. These demonstrated very clear changes to the 3D GaN multifacets depending on the deposition time of the *in-situ* SiN_x_ nanomask (see Supplementary Fig. S1). The evolution of the 3D GaN growth mode depended on the mask coverage, in line with a previous study using an *ex-situ* mask^18^. Finally, we fabricated three types of nanostructure templates by controlling the *in-situ* SiN_x_ nanomask density during MOCVD.

**Figure 3:**
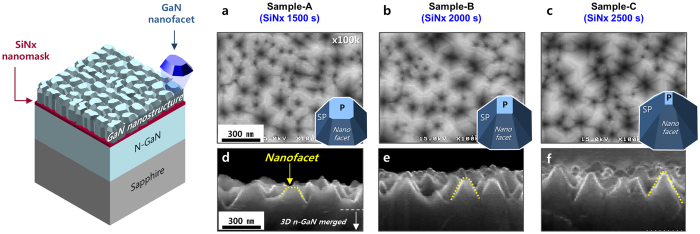
Schematic and SEM surface morphologies. The schematic indicates the nanostructure template with part of the GaN nanofacet. (**a**–**c**), (**d**–**f**) show SEM plane-view and cross-section view images, respectively of our three sample types as labeled. The insets in (**a**–**c**) indicate the schematics of the GaN nanofacets of each sample.

Figure 3 shows a schematic and the surface morphologies of these three n-GaN nanostructure templates. The schematic indicates the nanofacets (blue colour) on top of the 3D n-GaN nanostructures. Based on SEM analysis, the shape of the nanofacets on top of n-GaN nanostructure obviously changed depending on the SiN_x_ nanomask deposition time, described by the 3D GaN growth mechanism shown in Fig. 2. Each inset image shows the difference of the top surface shape of each GaN nanofacet and the facet ratio between P and SP for each. To measure the quantitative aspect ratio of the nanofacets of three templates, we also conducted the atomic forced microscopy (AFM) analysis.

**Figure 4:**
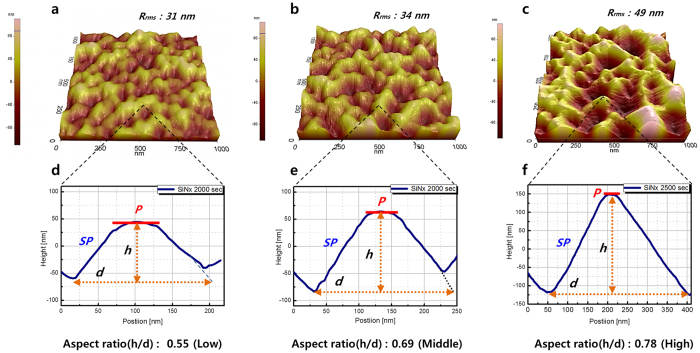
AFM surface morphologies. (**a**–**c**) Surface images with R_rms_ values of sample-A, sample-B, and sample-C, respectively. (scan area: 1 μm × 1 μm). (**d**–**f**) show AFM line scan profiles of each part of sample, indicating the differences of shape and aspect ratio (h/d) of each nanofacet.

Figure 4 shows AFM surface images of the nanofacets on each template; the root-mean-square roughness (R_rms_) of these three templates were 31 nm, and 34 nm, and 49 nm for sample-A, B, and C respectively. To clarify the change of aspect ratio among the three types of nanofacets, we measured AFM line scans on a representative nanostructure in each sample; the aspect ratios (h/d) were approximately 0.55(low), 0.69(middle), and 0.78(high), respectively. The differences between the facet ratios (P/SP) are clearly distinguished on each sample. The facet ratio is a very important factor for the adjusting the wavelength in PF-WLEDs using a 3D GaN multifacet technique^15^,^16^. Consequently we successfully fabricated various 3D n-GaN nanofacet templates using only *in-situ* SiN_x_ nanomasks.

### Indium composition analysis in the PF-WLED

To fabricate PF-WLEDs, we conducted the growth of LED structures on these n-GaN nanostructure templates with five pairs of InGaN/GaN MQWs and P-type GaN layers in order. All samples were simultaneously grown using the same growth conditions. Each LED was labelled as LED-A, LED-B, and LED-C, based on the previous n-GaN nanostructure templates sample-A, sample-B, and sample-C, respectively. To investigate the indium distribution in the MQWs, we conducted scanning transmission electron microscopy (STEM) and energy-dispersive x-ray spectroscopy (EDS) on LED-A (sample-A: 1500 sec) and LED-C (sample-C: 2500 sec).

**Figure 5:**
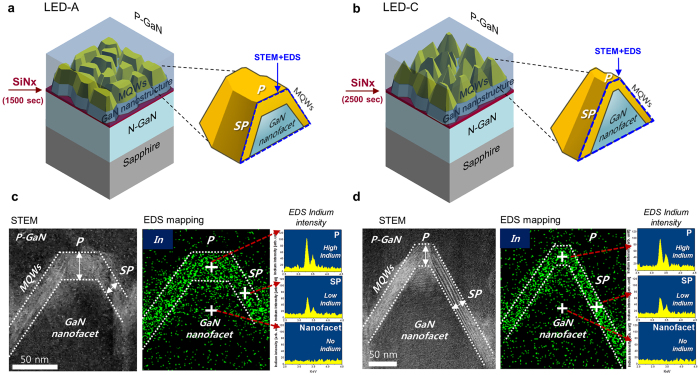
Cross-sectional STEM-EDS characterization. (**a**,**b**) Schematics of each LED structure and each nanofacet. (**c**,**d**) Cross-sectional STEM-EDS mapping images and EDS indium spot spectra of LED-A and LED-B, respectively.

Figure 5(a,b) show a schematic of two LEDs and the analysis position for each nanofacet. LED-A and LED-C consisted of different nanofacet structures with low aspect ratio and high aspect ratio, respectively. Figure 5(c,d) show the STEM, EDS-mapping, and EDS spot results of the MQWs in each LED structure, respectively. The STEM and EDS-mapping images show that the InGaN/GaN MQWs were grown with different thicknesses on both the P and SP planes of the n-GaN nanofacets, indicated with white arrows in each STEM image. Moreover, the indium EDS spot intensity and spectrum on each facet in both samples obviously indicate that the indium incorporation in the top plane (P) is nearly twice as high as in inclined-plane (SP), and there is no indium intensity at the GaN nanofacet. This is comparable to previous reports that utilize as *ex-situ* multifacet method^11^. It was reported previously that the diffusivity of indium adatoms on the SP-plane is larger than that on the P-plane. The surface energy on the P-plane is lower than that of the SP-plane because the SP-plane consisted of nitrogen terminated sites, whereas the P-plane is terminated with gallium sites; therefore, more indium adatoms migrate into the P-plane. Moreover, the diffusivity of gallium atoms is also larger at the SP-plane than the P-plane, similar to indium atoms as mentioned previously. As a result, the total thickness of the active region on the P-plane is larger than that of SP^11^,^19^. Therefore, InGaN QW thickness and indium composition depend on the polarity of the 3D GaN nanofacet. From these results, the low aspect ratio nanofacet LED (LED-A) has more in-rich sites (P-plane) than that of the high aspect ratio nanofacet LED (LED-C).

**Figure 6:**
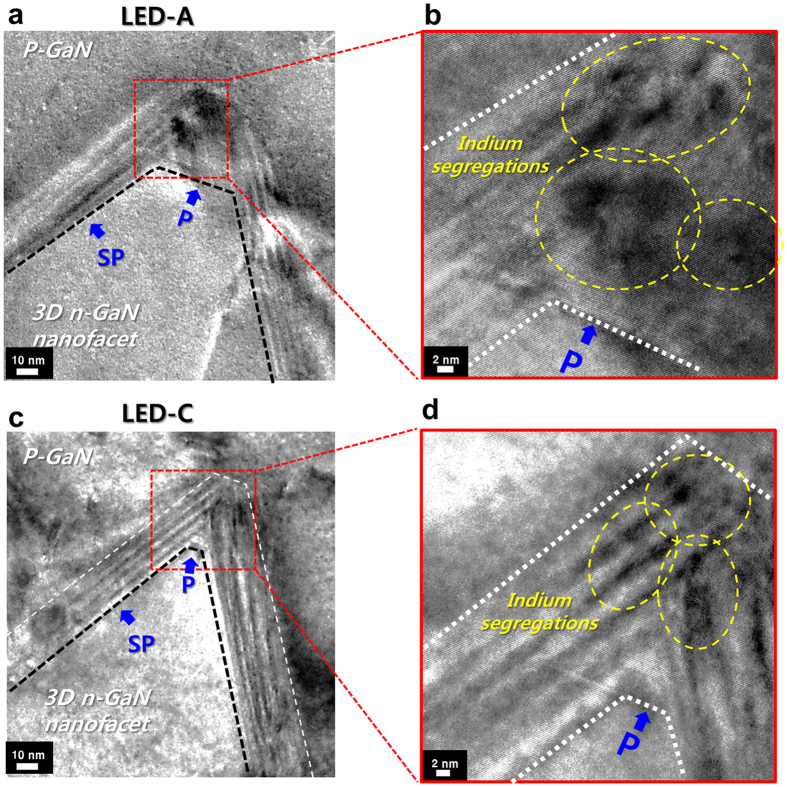
Cross-sectional TEM characterization [z = [1–100]]. (**a**,**b**) and (**c**,**d**) show the MQWs on nanofacets (on SP-planes) and indium segregation regions (on P-planes) of LED-A and LED-C, respectively.

Additionally, the cross-sectional TEM analysis was also performed to confirm the presence of indium segregation at each crystal plane (P-plane and SP-plane) of the nanofacet. As shown in Fig. 6(a–d), both LED-A and LED-C show the indium segregation regions on the P-planes. These indium segregations on the P-planes are very similar to the results of previous studies^10^,^20^,^21^. However, it is important to note that segregation area of LED-A (low aspect ratio) is wider than that of LED-C (high aspect ratio). On the other hand, indium segregation on the SP plane was hardly observed in both samples. These characteristics are consistent with the results in Fig. 5(c,d).

### Optical characteristics of the nanofacet-controlled PF-WLEDs

**Figure 7:**
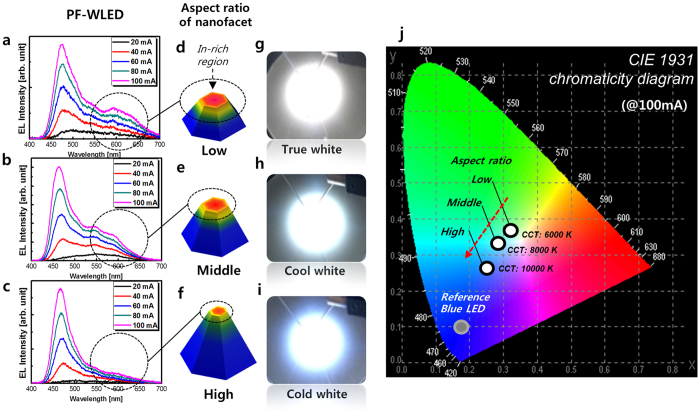
EL spectra of three LEDs. (aspect ratio variation). (**a**–**c**) EL spectra of LED-A (low aspect ratio), LED-B (middle aspect ratio), and LED-C (high aspect ratio), respectively. (**d**–**f**) Schematic of the wavelength origin on each nanofacet. (**g**–**i**) EL emission images of LED-A, LED-B, and LED-C, respectively. (**j**) Colour coordinate and T_c_ according to the EL spectra of LED-A, LED-B, and LED-C on the Commission Internationale de 1’Eclairage (CIE) 1931 chromaticity diagram. T_c_ increases as the aspect ratio of nanostructure in the PF-WLED increases.

**Figure 8:**
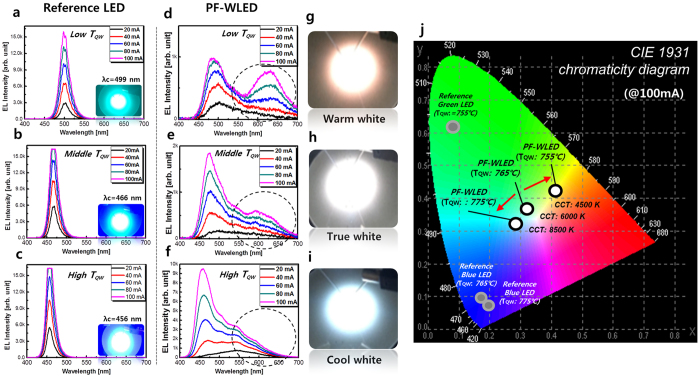
EL spectra of three LEDs. (QW growth temperature variation). (**a**–**c**) EL spectra and emission images of three reference LEDs as functions of QW growth temperature (755 °С, 765 °С, and 775 °С respectively). (**d**–**f**) and (**g**–**i**) EL spectra and emission images of three PF-WLEDs, respectively. All PF-LEDs are based the low aspect ratio structure, but have different QW growth temperatures listed previously. (**j**) Colour coordinate and T_c_ based on EL spectra of T_QW_ low PF-WLED, T_QW_ middle PF-WLED, and T_QW_ high PF-WLED on the Commission Internationale de 1’Eclairage (CIE) 1931 chromaticity diagram. Each colour coordinate of the reference LEDs is also indicated in the diagram. T_c_ depends on the T_QW_ variation of the nanostructures in PF-WLEDs.

Figure 7 shows the EL results of three PF-WLEDs at the injection current range of 20–100 mA. The reference LED’s peak wavelength (λ_p_) is 466 nm (blue emission) at 100 mA with a narrow width, as indicated in Fig. 8(b). As shown in Fig. 7(a–c), the spectra of all three LEDs show broad emissions caused by indium fluctuations in the MQWs grown on their nanofacets. EL spectra of these LEDs also show more intense blue emissions (λ_p_: 435–490 nm) than that of the longer wavelength region (λ_p_: 520–650 nm) because the nanofacets have more SP-planes (low indium incorporation) than the P-planes (high indium incorporation), as shown in both Figs 4 and 5. Figure 7(a–c) show the obvious differences of in the long wavelength regions (λ_p_: 570–660 nm) in the EL spectra of the three LEDs, marked by dotted line-circles and described in Fig. 7(d–f). These wavelength variations, which are dependent on the multifacets, were also reported in *ex-situ* multifacet structures^12^,^16^. The EL images of each LED at an injection current of 100 mA are shown in Fig. 7(g–i). The T_c_ of each PF-WLED changed with the aspect ratio of the 3D nanofacet. Their colour coordination is indicated using the Commission Internationale de 1’Eclairage (CIE) 1931 chromaticity diagram shown in Fig. 7(j). The T_c_ of the three LEDs was also changed from 6000 K (true white) to 10000 K (cold white), demonstrating that T_c_ control is possible by *in-situ* nanofacet engineering.

### Optical characteristics of the QW temperature-controlled PF-WLED

It is well known that indium composition in MQWs crucially depends on the growth temperature. So we tried to change MQW’s growth temperature only using LED-A structure (low aspect ratio PF-WLED: true white emission). The EL results are shown in the Fig. 8. Figure 8(a–c) and (d–f) show the EL spectra of three reference LEDs and three PF-WLEDs. The quantum well growth temperature (T_QW_) of each reference LED is 755 °С (low T_QW_), 765 °С (middle T_QW_), and 775 °С (high T_QW_), respectively. As mentioned previously, all PF-WLEDs have the same nanofacet structure as LED-A [low aspect ratio PF-WLED in Fig. 7(d)]. As shown in Fig. 8(a–c), the centre wavelengths (λ_c_) of each reference LED is 499 nm (green), 466 nm (blue), and 456 nm (blue), respectively at an injection current of 100 mA. The indium incorporation rate in InGaN QWs is also inversely proportional to T_QW_. The EL spectrum of the low T_QW_ reference LED (Fig. 8(a)) shows the lowest optical intensity and the broadest full width at half maximum (FWHM) among the three reference LEDs. It is likely that there are a few indium segregation sites in InGaN QWs caused by partial strain relaxation due to the high indium incorporation at low T_QW_. On the other hand, the EL spectrum of the high T_QW_ reference LED (Fig. 8(c)) shows the highest optical intensity and narrowest FWHM value. Similar results are also observed in the PF-WLEDs. Figure 8(d–f) show the T_QW_ controlled white spectra of three PF-WLEDs. The EL spectrum of the low T_QW_ PF-WLED shows the highest intensity in the long wavelength region (λ_p_: 570–660 nm) compared to those of the middle and high T_QW_ PF-WLEDs. Moreover, the λ_c_ of the short wavelength region is 488 nm, which is longer than those of the other LEDs due to high indium incorporation at low T_QW_. Both the short and long spectral regions show blue-shifts of the peak wavelength with increasing injection current level, which indicates the band filling effect of the local potential minimum in the potential fluctuation of the In-rich InGaN well layer^22^. On the other hand, the EL spectrum of high T_QW_ PF-WLED shows the lowest intensity in the long wavelength region compared to that of the low and middle T_QW_ PF-WLEDs. Moreover, λ_c_ is 459 nm in short wavelength region here, the shortest among the three PF-WLEDs due to the low indium incorporation rate in InGaN QWs at high T_QW_. Figure 8(d–f) show the λ_c_ variation in the short wavelength region by T_QW_ was similar to that of the reference LEDs, whereas the variations in the long wavelength based on T_QW_ was more remarkable because the indium incorporation rate of In-rich QWs on the narrow top region (P-plane) is more sensitive in regards to T_QW_ than the SP-plane. EL images of each PF-LED at an injection current of 100 mA are shown in Figure 8(g–i), and their colour coordination is indicated by the Commission Internationale de 1’Eclairage (CIE) 1931 chromaticity diagram shown in Fig. 8(j). As shown here, T_c_ of the low T_QW_ PF-WLED decreased to 4500 K, compared to that of the middle T_QW_ PF-WLED (6000 K) due to the high intensity in the long wavelength region (Fig. 8(d)). On the other hand, T_c_ of the high T_QW_ PF-WLED increased to 8500 K due to the dominant short wavelength spectral region of the low T_QW_ PF-WLED. These results indicate that T_c_ of the PF-WLEDs could be controlled from the warm white region to the cool white region by changing the T_QW_ condition on the 3D GaN nanostructures in PF-WLEDs.

The results also showed the obvious difference of light intensity between reference blue LED and suggested PF-WLEDs (see Supplementary Fig. S3). However, it is notable that the light intensity of LED-A (T_QW_: 775 °C) was significantly higher than those of other proposed PF-WLEDs, which suggest the possibility of light intensity enhancement of proposed PF-WELDs through further research.

## Conclusions

We demonstrated colour-crafted PF-WLEDs using *in-situ* nanostructure engineering for the first time. Here we used only conventional blue or green wavelength QW growth conditions to change the white colour-temperature in our *in-situ* nanofaceted PF-WLEDs. This can increase the commercialization potential of our approach as most similar systems require more complicated growth techniques to protect against degradation of the In-rich InGaN QW region. Our technology uses a commercial MOCVD system without external photolithography for an *in-situ* one-step growth process to control the T_c_ of the white emission in PF-WLEDs. Therefore, this practical method has a low technical barrier. Moreover, there was no wafer contamination due to the lack of *ex-situ* patterning. We believe that this *in-situ* method can be applied to large-diameter wafers to fabricate PF-WLEDs with various T_c_ values (see Supplementary Fig. S2). Further work in necessary for improving optical performance, and is planned as future research. In this study, we preferentially focused on the possibility of T_c_ control of PF-WLEDs using our new approach. Finally, we expect that this *in-situ* nanostructure variation technique with *in-situ* SiN_x_ nanomask control will be useful for a variety of nanotechnology applications.

## Methods

### Growth of n-GaN nanostructure templates and LEDs

We used a Aixtron close-coupled showerhead (CCS-MOCVD) reactor system to grow 3D n-GaN nanostructure with SiNx nanomask and LEDs. After annealing the sapphire substrate in H_2_ at 1080 °С for 5 min, a 30-nm-thick GaN buffer layer was deposited by MOCVD at 550 °С, followed by growth of a 2-μm-thick undoped GaN(u-GaN) and 4-μm-thick n-type GaN(n-GaN) layers at 1060 °С in order. After bringing the temperature down to 860 °С, a SiN_x_ nanomask layer was deposited on the n-GaN template in H_2_ for 1500 s, 2000 s, and 2500 s, followed by the growth of a about 0.6~0.8-μm-thick 3D n-GaN structure at 850 °С. An InGaN/GaN MQW structure was grown on the template with an InGaN thickness of 2 nm and a GaN thickness of 5 nm. Finally, 0.25-μm-thick p-type GaN cladding layer were grown by MOCVD. Trimethylindium (TMIn), trimethylgallium (TMGa), silane (SiH_4_) and ammonia (NH_3_) were used as the precursors for indium, gallium, silicon and nitrogen, respectively. Silane (SiH_4_) and bis(cyclopentadienyl) magnesium (Cp_2_Mg) were used as n-type and p-type dopant sources, respectively.

### Characterizations

The surface morphology of the samples was measured by SEM (Hitach-4000) and AFM (XE-1500, Park system). The cross-sectional microstructure and distribution of the indium concentration of the samples were analyzed by TEM (JEM-2100F, JEOL, 300 KeV) and EDS with STEM (JEM-2100F, JEOL, 200 KeV and Cs corrector, CEOS), respectively. The STEM specimens were prepared by focused ion beam system (NOVA 600 Nanolab). Also EL (ELT-1100 LED Chip tester) was used to determine the optical properties at 300 K of the InGaN based reference LEDs and PF-WLED LEDs.

## Additional Information

**How to cite this article**: Min, D. *et al*. Colour-crafted phosphor-free white light emitters via *in-situ* nanostructure engineering. *Sci. Rep.* **7**, 44148; doi: 10.1038/srep44148 (2017).

**Publisher's note:** Springer Nature remains neutral with regard to jurisdictional claims in published maps and institutional affiliations.

## Supplementary Material

Supplementary Data

## References

[b1] Schubert E. F. & Kim J.-K. Solid-state light sources getting smart. Science 308, 1274–1278 (2005). 15919985 10.1126/science.1108712

[b2] Kim J.-K. & Schubert E. F. Transcending the replacement paradigm of solid-state lighting. Opt. Express 16, 21835–21842 (2008). 19104616 10.1364/oe.16.021835

[b3] Smet P. F. & Parmentier A. B. & Poelman Selecting conversion phosphors for white light-emitting diodes. J. Electrochem. Soc. 158, R37–R54 (2011).

[b4] Kimura N., Sakuma Ken., Hirafune S. & Asano K. Extrahigh color rendering white light-emitting diode lamps using oxynitride and nitride phosphors excited by blue light-emitting diode. Appl. Phys. Lett. 90, 051109 (2007).

[b5] Meneghesso G., Meneghini A. & Zanoni E. Recent results on the degradation of white LEDs for lighting. J. Phys. D Appl. Phys. 43, 354007 (2010).

[b6] Damilano B., Grandjean N., Pernot C. & Masseis J. Monolithic white light emitting diodes based on InGaN-GaN multiple-quantum wells. Jpn. J. Appl. Phys. 40, L 918–L 920 (2001).

[b7] Yamada M., Narukawa Y. & Mukai T. Phosphor free high-luminous-efficiency white light-emitting diodes. Jpn. J. Appl. Phys. 41, L 246–L 248 (2002).

[b8] Shei S. C. . Emission mechanism of mixed-color InGaN-GaN multi-quantum-well light-emitting diode. Jpn. J. Appl. Phys. 45, 2463–2466 (2006).

[b9] Wang X. H. . White light-emitting diodes based on a single InGaN emission layer. Appl. Phys. Lett. 91, 161912 (2007).

[b10] Li H. . Phosphor-free, color-tunable monolithic InGaN light-emitting diodes. Appl. Phys. Express 6, 102103 (2013).

[b11] Funato M. . Tailored emission color synthesis using microfacet quantum wells consisting of nitride semiconductors without phosphors. Appl. Phys. Lett. 88, 261920 (2006).

[b12] Funato M. . Monolithic polychromatic light-emitting diodes based on InGaN microfacet quantum wells toward tailor-made solide-state lighting. Appl. Phys. Express 1, 011106 (2008).

[b13] Yang G. . White-light emission from InGaN-GaN quantum well microrings grown by selective area epitaxy. Photon. Res. 4, 17–20 (2016).

[b14] Wu K. . Fabrication and optical characteristics of phosphor-free InGaN nanopyramid white light emitting diodes by nanospherical-lens photolighgraphy. J. Appl. Phys. 115, 123101 (2014).

[b15] Ueda M. . Additive color mixture of emission from In Ga N Ga N quantum wells on structure controlled GaN microfacets. Appl. Phys. Lett. 90, 171907 (2007).

[b16] Funato M. . Emission color tunable light-emitting diodes composed of InGaN multifacet quantum wells. Appl. Phys. Lett. 93, 021126 (2008).

[b17] Min D. H., Park D. H., Jang J. J., Lee K. S. & Nam O. H. Phsphor-free white-light emitters using *in-situ* GaN nanostructures grown by metal organic chemical vapor deposition. Sci. Rep. 5, 17372 (2015). 26626890 10.1038/srep17372 PMC4667288

[b18] Lundskog A. . Controlled growth of hexagonal GaN pyramids by hot-wall MOCVD. J. Cryst. Growth 363, 287–293 (2013).

[b19] Ko Y. H., Song J. E., Leung B., Han J. & Cho Y. H. Multi-color broadband visible light source via GaN heagonal annular structure. Sci. Rep. 4, 05514 (2014). 10.1038/srep05514 PMC4076692 24981889

[b20] Wang T. C. . Study of InGaN Muliple Quantum Dots by Metal Organic Chemical Vapor Deposition. Jpn. J. Appl. Phys. 45, 3560–3563 (2006).

[b21] Wang X. H. . White light-emitting diodes based on a single InGaN emission layer. Appl. Phys. Lett. 91, 161912 (2007).

[b22] Nakamura S. The roles of structural imperfections in InGaN-based blue light-emitting diodes and laser diodes. Science 281, 956–961 (1998). 10.1126/science.281.5379.956 9703504

