# Supplementary material for: Colour-crafted phosphor-free white light emitters via in-situ nanostructure engineering
Source: Sci Rep. 2017 Mar 8;7:44148. doi: 10.1038/srep44148 (PMC5341162; doi:10.1038/srep44148)
Supplement: Supplementary Data [file srep44148-s1.doc]

Supplementary Data

Colour-crafted phosphor-free white light emitters via in-situ nanostructure engineering

Daehong Min, Donghwy Park, Kyuseung Lee, and Okhyun Nam*

Convergence Center for Advanced Nano Semiconductors (CANS), Department of Nano-Optical Engineering, Korea Polytechnic University, 237, Sangidaehak-ro, Siheung-si, Gyeonggi-do 429-793, South Korea

E-mail: ohnam@kpu.ac.kr


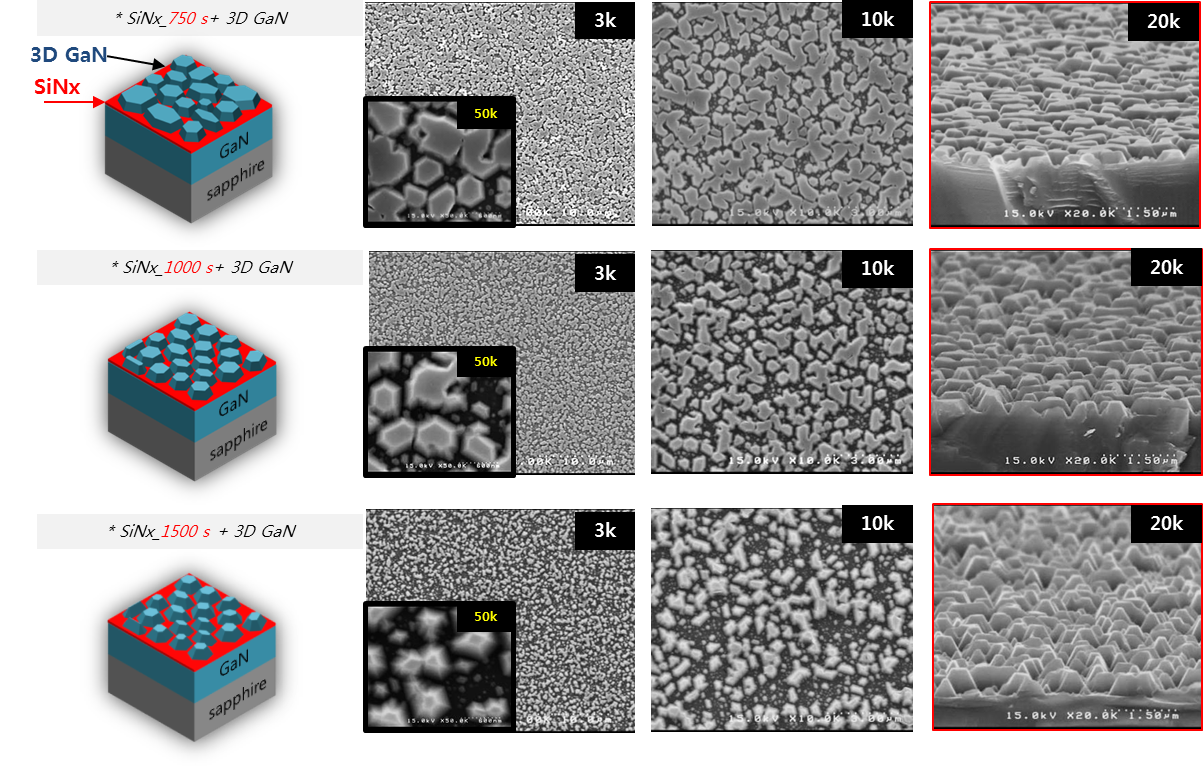


**Supplementary Fig. S1│ Schematic and SEM surface morphologies of the regrown 3D GaN.** These schematics indicate the facet development of nanostructures based on deposition time (750 s, 1000 s, and 1500 s, as labelled). The growth temperature and time to obtain regrown GaN is identical for all templates (970 °С and 300 s). The plan-view and bird’s-eye SEM images show the obvious shape change of 3D GaN nanofacets due to the opening density variation of the in-situ SiN_x_ nanomask.


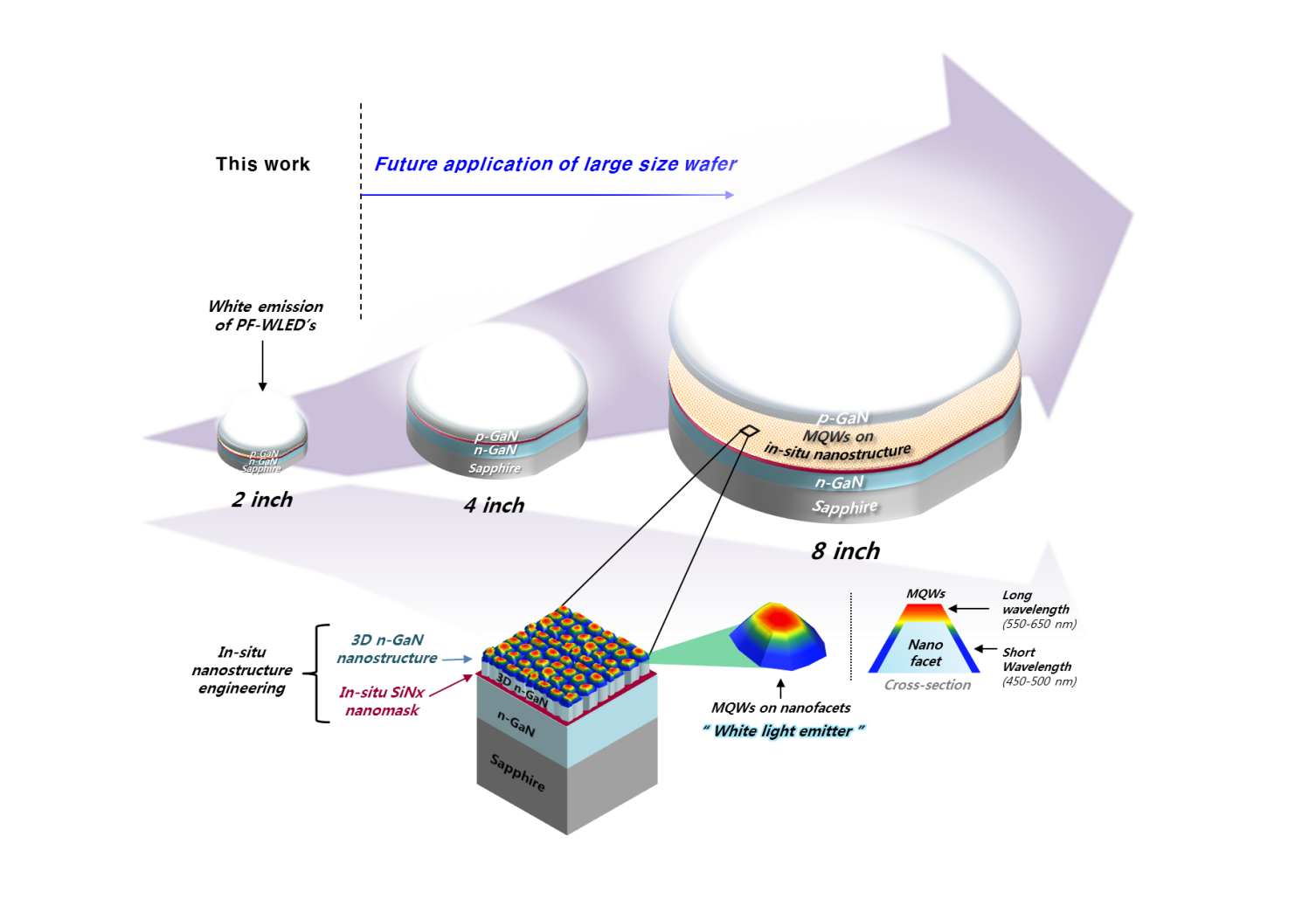


**Supplementary Fig. S2│ Schematic of large diameter PF-WLEDs.** The schematic indicates the possibility of application using our in-situ nanotechnology involving an in-situ SiN_x_ nanomask and 3D-GaN nanostructuring without any external photolithography. This technology can be utilized at low cost and large-scale wafers for future commercialization.


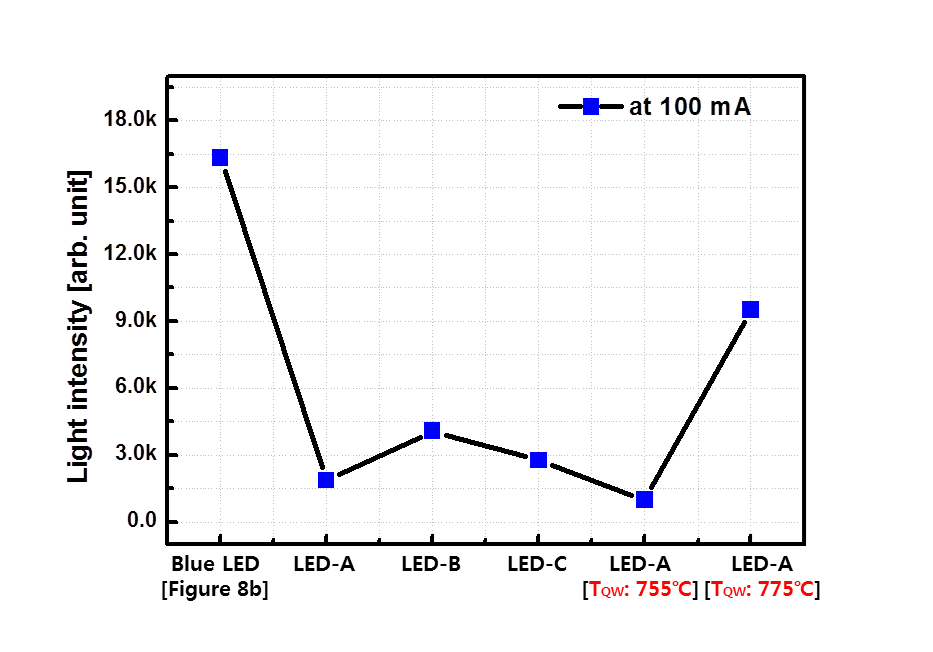


**Supplementary Fig. S3│ Graph of light intensity of PF-WLEDs and blue LED.** The graph indicates the comparison of light intensities between blue LED (Fig. 8b) and PF-WLEDs (Fig. 7a-c and Fig.8d-f).
